# ElemeNT 2023: an enhanced tool for detection and curation of core promoter elements

**DOI:** 10.1093/bioinformatics/btae110

**Published:** 2024-02-24

**Authors:** Orit Adato, Anna Sloutskin, Hodaya Komemi, Ian Brabb, Sascha Duttke, Philipp Bucher, Ron Unger, Tamar Juven-Gershon

**Affiliations:** The Mina and Everard Goodman Faculty of Life Sciences, Bar-Ilan University, Ramat Gan, 5290002, Israel; The Mina and Everard Goodman Faculty of Life Sciences, Bar-Ilan University, Ramat Gan, 5290002, Israel; The Mina and Everard Goodman Faculty of Life Sciences, Bar-Ilan University, Ramat Gan, 5290002, Israel; School of Molecular Biosciences, College of Veterinary Medicine, Washington State University, Pullman, WA 99164, United States; School of Molecular Biosciences, College of Veterinary Medicine, Washington State University, Pullman, WA 99164, United States; Swiss Institute of Bioinformatics (SIB), 1015 Lausanne, Switzerland; The Mina and Everard Goodman Faculty of Life Sciences, Bar-Ilan University, Ramat Gan, 5290002, Israel; The Mina and Everard Goodman Faculty of Life Sciences, Bar-Ilan University, Ramat Gan, 5290002, Israel

## Abstract

**Motivation:**

Prediction and identification of core promoter elements and transcription factor binding sites is essential for understanding the mechanism of transcription initiation and deciphering the biological activity of a specific locus. Thus, there is a need for an up-to-date tool to detect and curate core promoter elements/motifs in any provided nucleotide sequences.

**Results:**

Here, we introduce ElemeNT 2023—a new and enhanced version of the Elements Navigation Tool, which provides novel capabilities for assessing evolutionary conservation and for readily evaluating the quality of high-throughput transcription start site (TSS) datasets, leveraging preferential motif positioning. ElemeNT 2023 is accessible both as a fast web-based tool and via command line (no coding skills are required to run the tool). While this tool is focused on core promoter elements, it can also be used for searching any user-defined motif, including sequence-specific DNA binding sites. Furthermore, ElemeNT’s CORE database, which contains predicted core promoter elements around annotated TSSs, is now expanded to cover 10 species, ranging from worms to human. In this applications note, we describe the new workflow and demonstrate a case study using ElemeNT 2023 for core promoter composition analysis of diverse species, revealing motif prevalence and highlighting evolutionary insights. We discuss how this tool facilitates the exploration of uncharted transcriptomic data, appraises TSS quality, and aids in designing synthetic promoters for gene expression optimization. Taken together, ElemeNT 2023 empowers researchers with comprehensive tools for meticulous analysis of sequence elements and gene expression strategies.

**Availability and implementation:**

ElemeNT 2023 is freely available at https://www.juven-gershonlab.org/resources/element-v2023/. The source code and command line version of ElemeNT 2023 are available at https://github.com/OritAdato/ElemeNT. No coding skills are required to run the tool.

## 1 Introduction

Successful analysis of gene regulation depends upon knowledge of underlying DNA sequence motifs. To understand the factors and mechanisms mediating expression of a given gene of interest, it is thus critical to accurately define the underlying DNA sequence motifs. The core promoter, often referred to as “the gateway to transcription” (Heintzman and Ren 2007, Juven-Gershon *et al.* 2008), is an 80-bp region that may contain one or more short DNA sequences, termed core promoter elements/motifs. These elements and their composition are central in the process of transcription initiation by RNA polymerase II (Pol II) (Thomas and Chiang 2006, Ohler and Wassarman 2010, Wang *et al.* 2014, Danino *et al.* 2015, Haberle and Lenhard 2016, Vo Ngoc *et al.* 2019, Sloutskin *et al.* 2021). Notably, many sequence-specific transcription factors (TFs) bind to the proximal promoter region (within ∼ −150 to −50, relative to the TSS). Together, the spacing and composition of core promoter elements and TFs play an important role in the spatio-temporal pattern of Pol II initiation (Lenhard *et al.* 2012, Spitz and Furlong 2012, Lu *et al.* 2020).

ElemeNT (Sloutskin *et al.* 2015) is a tool used for detection and curation of core promoter elements within user-provided sequences. It utilizes position weight matrices generated based on experimentally validated sequences, rather than over-represented motifs. This web-based interactive tool can be easily used to predict and display putative core promoter elements and their biologically relevant combinations. Here, we present a new and advanced version, ElemeNT 2023, that enables researchers to identify core promoter elements, transcription factor binding sites (TFBSs) or any DNA sequence motif of interest, examine their evolutionary conservation across species, and obtain valuable biological insights. In addition, ElemeNT 2023 facilitates a unique approach to rapidly assess the quality of high-throughput TSS datasets leveraging natural spacing preferences of diverse DNA sequence motifs.

## 2 Materials and methods

ElemeNT 2023 is implemented in Perl, and the installation package of the command line version is available for download in GitHub. This version was extended to include four new core promoter elements, namely pause button (PB), the BBCA_+1_BW initiator, Ohler Motif 1 (Motif 1) and GAGA factor binding site. The position weight matrices (PWMs) of the PB, BBCA_+1_BW initiator and Motif 1 are based on published experimental data (Ohler *et al.* 2002, Hendrix *et al.* 2008, Vo Ngoc *et al.* 2017). The PWM of the GAGA factor binding site is based on CISBP motif M5247, version 1.02 (Weirauch *et al.* 2014).

### 2.1 Normalization to GC content

The fraction of the provided GC is divided by 2 to represent the background probabilities of the individual occurrences of G and C. Accordingly, the background probabilities of individual occurrences of A and T were calculated as (1 − GC)/2. These probabilities were used to normalize the PWM of the correlated nucleotides in the motif, i.e. the nucleotide score at every position in the PWM was calculated as follows: in case of G or C score_(__*i*__,_*_j_*_)_ = log_2_(*P*_(__*i*__,__*j*__)_/*P*_GC/2_), and in case of A or T it was calculated as score_(__*i*__,__*j*__)_=log_2_(*P*_(__*i*__,__*j*__)_/*P*_(1−GC)/2_).

### 2.2 Visualizing the distributions of core promoter elements among nascent transcription peaks

We have recently performed nascent RNA sequencing using capped-small RNA sequencing (csRNA-seq) (Duttke *et al.* 2019) of the first 8 h of *Drosophila melanogaster* embryonic development. Data are available at Gene Expression Omnibus (GEO) accession number GSE221852. csRNA-seq data (GSE135498) of human K562 cells and mouse bone marrow-derived macrophages (BMDM) (Duttke *et al.* 2019) were also analyzed. Peak calling was performed using HOMER (Duttke *et al.* 2019). For the identified annotated transcription start regions, the genomic sequences of the core promoter region (±100 bp around TSS) were downloaded. These sequences were used as input to ElemeNT 2023 for detection of the TATA box (TATA), *Drosophila* initiator (dInr), PB and downstream core promoter element (DPE), GAGA, motif 1 and dTCT motifs. For each searched element, we used a python script to calculate the fraction (%) of transcripts containing the element identified by ElemeNT at a specific position (out of a total number of the specific element), and their median score in the specific position. The graphs were generated using a custom R script (available in GitHub).

## 3 Results

### 3.1 ElemeNT workflow

**Figure 1.**
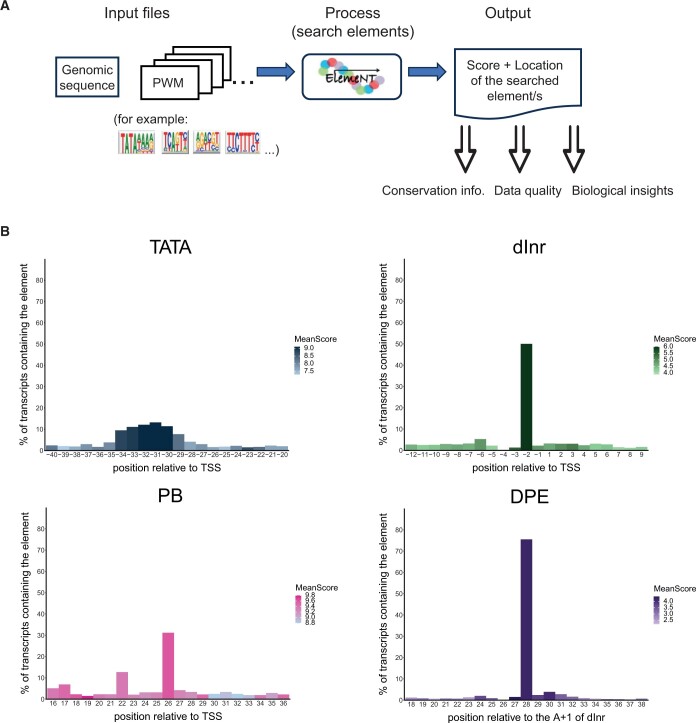
(A) General workflow of analysis with ElemeNT 2023. Sequences and PWMs of core promoter elements are provided as input to the ElemeNT utility. The output of the utility run includes the location and score for every detected element. (B) Results of ElemeNT 2023 run on TSSs derived from nascent csRNA-seq of the first 8 h of *D. melanogaster* embryonic development. The *X*-axis represents the predicted element position relative to the TSS for the TATA box, dInr and PB, and the predicted position relative to the A_+1_ of the dInr for the DPE. The *Y*-axis represents the percentage of transcripts containing the element at the specific position (out of all transcripts containing the element). The color hue represents the mean score of the predicted elements within a specific position, as indicated in the color legend.

ElemeNT detects and annotates core promoter elements (or any user-defined TFBS or sequence motif), and calculates its PWM log score (higher or equal to the defined cutoff; Fig. 1A). The output can be used as a quality control tool for transcriptomics datasets and to gain new biological insights into examined data. For example, the quality of the input data and the sequence conservation of the elements can be evaluated based on the distribution and scores of a DNA motif occurrence relative to the reported TSSs.

Specifically, the new functionality added to ElemeNT 2023 includes:

1. An updated web-based user interface that includes UCSC sessions (vCORE; https://www.juven-gershonlab.org/resources/vcore/) for visualization of detected elements in ten species, from worm to human
2. PWMs search scores are calculated using log likelihoods: The PWMs used for motif search in ElemeNT 2023 are now calculated as log likelihoods (log_2_), providing higher resolution for small-scale differences. PWMs can be downloaded as an excel table from https://www.juven-gershonlab.org/resources/element-v2023/element-v-2023-manual/
3. Newly added features:
  - normalization to GC-content;
  - elements search can be performed in both directions (i.e. sense and antisense DNA strands); and
  - the current collection of experimentally validated core promoter elements was expanded to include: the human BBCA_+1_BW initiator, as well as three motifs that were previously implicated in Pol II pausing, namely Motif 1, Pol II PB and the GAGA factor binding site (Li and Gilmour 2013).
4. An extended capability for quality control analysis of next-generation sequencing transcriptomic data based on spacing of sequencing motifs relative to the TSSs. Following the suggested workflow (Fig. 1A), the output of ElemeNT 2023 can be used as input to the script available in GitHub.

### 3.2 Case study for ElemeNT 2023 usage

#### 3.2.1 Application of ElemeNT 2023 to nascent RNA data

In the case study presented in this applications note, ElemeNT 2023 was applied to genomic sequences around experimentally determined nascent TSSs captured by csRNA-seq (Duttke *et al.* 2019) during the initial 8 h of *D. melanogaster* embryonic development (Fig. 1B). The analysis revealed the conservation of positional information and motif score, inferring biological significance that can be utilized to assess the quality of high-throughput TSS datasets. For instance, the majority of identified dInr and DPE motifs were precisely positioned relative to the TSS, specifically at −2 and +28, respectively. This aligns with the strict spacing dependency of the DPE on the dInr motif (Burke and Kadonaga 1997, Kutach and Kadonaga 2000). The observed elevated mean scores (indicated by the darker color in Fig. 1B) of motifs occurring at the expected positions, further underscore the requirement of exact positioning of these motifs for their biological function.

In contrast, TATA box sequences exhibited broader spatial variability, in line with previous studies (Ohler *et al.* 2002, Gershenzon and Ioshikhes 2005, Carninci *et al.* 2006, Ponjavic *et al.* 2006). Notably, while the PB motif's location lacks strict adherence (Hendrix *et al.* 2008), the most prevalent PB position was downstream [in line with Hendrix *et al.* (2008)], with a marked preference at +26. In contrast, the GAGA factor binding site, which was also implicated in Pol II pausing motif (Li and Gilmour 2013) is not strictly positioned (Supplementary Fig. S1). Both Motif 1 and dTCT were strictly localized, with peaks at −5 and −2 upstream of the TSS (Supplementary Fig. S1). Analysis of available csRNA-seq data (GSE135498) of human K562 cells and mouse bone marrow-derived macrophages (BMDM) (Supplementary Fig. S2) demonstrates that the use of ElemeNT 2023 is not limited to *D. melanogaster*. These findings underscore ElemeNT's capacity to precisely detect and characterize core promoter elements within their relevant genomic context, shedding light on their distribution and conservation.

#### 3.2.2 The CORE database was expanded to 10 species, from worm to human

The ElemeNT CORE database, which contains predicted core promoter elements around Cap Analysis of Gene Expression (CAGE)-annotated TSSs (https://epd.expasy.org/epd/EPDnew_database.php), was expanded from *Drosophila* to now include 10 species, ranging from worm to human (*D. melanogaster* (dm6), *Homo sapiens* (hg38), *Macaca mulatta* (rheMac8), *Mus musculus* (mm10), *Rattus norvegicus* (rn6), *Gallus gallus* (galGal5), *Canis familiaris* (canFam3), *Apis mellifera* (amel5), *Danio rerio* (danRer7), and *Caenorhabditis elegans* (ce6)). This analysis is available at https://www.juven-gershonlab.org/resources/core-2023/ as a precompiled file named CORE.

Comparing promoter sequences from these species (Dreos *et al.* 2015, Meylan *et al.* 2020) revealed that the positions of both the TATA box and the dInr motif are largely conserved among these species (Supplementary Fig. S3). However, the mean score of dInr is higher in fly and honeybee, compared to the other species analyzed. The PB motif, which was first discovered in flies (Hendrix *et al.* 2008) seems to be fly-specific (Supplementary Fig. S3). While it was previously detected between +20 and +30 (Hendrix *et al.* 2008), both nascent transcriptomics and EPDnew indicate its enrichment in position +26 in *D. melanogaster* (Fig. 1B and Supplementary Fig. S3).

Overall, both the location and the scores of the detected motifs were similar across the species, indicating the functionality of these elements is conserved across the diverse analyzed metazoan species. The updated CORE database is available at https://www.juven-gershonlab.org/resources/core-2023/. UCSC visual sessions (vCORE) are available at https://www.juven-gershonlab.org/resources/vcore/.

#### 3.2.3 ElemeNT 2023 as a tool to evaluate high-throughput transcriptomics datasets and transcription start site quality

Many core promoter elements exhibit strict functional spacing dependencies on the TSS. ElemeNT 2023 quickly determines core promoter element distributions relative to the TSS, enabling assessing TSS quality based on biological properties. In this case study, we compared the EPDnew data (Supplementary Fig. S3), which is largely based on CAGE, with nascent csRNA-seq data (Fig. 1), and RNA annotation and mapping of promoters for the analysis of gene expression (RAMPAGE) data of *D. melanogaster* (Supplementary Fig. S4, GSE89299; Batut and Gingeras 2017). This comparison revealed the spatial preference of the dInr, PB and DPE as most stringent in csRNA-seq captured nascent TSSs, while the RAMPAGE-based TATA box seems more strictly positioned than the TATA box identified in the csRNA-seq data.

Together, this case study demonstrates the use of ElemeNT and biological features, such as core promoter element spacing constraints, to evaluate high-throughput transcriptomics data.

## 4 Discussion

Predicting and identifying core promoter elements and TFBSs is an essential part in the process of understanding the mechanism of transcription initiation and deciphering the biological activity of a specific locus. ElemeNT 2023 is a simple, fast, and user-friendly web-based interactive tool for prediction and display of any sequence element. It is also available as a command line tool on GitHub. ElemeNT 2023 was designed to annotate these elements in any combination and genomic sequence. In addition to the location and score of identified elements, it also displays their biologically relevant combinations (e.g. the dependency of downstream core promoter elements on the presence of an Inr motif and the precise spacing from it), without a need for prior determination of the TSS. Notably, given the spatial preference of many DNA sequence motifs relative to the TSSs (Fig. 1) (Delos Santos *et al.* 2022), ElemeNT 2023 also provides a unique approach to assess the quality of high-throughput TSS datasets and to confirm the existence of a core promoter region.

ElemeNT 2023 includes the functionality of searching for any user-provided PWM in a given sequence. It can also be used for predicting core promoter elements and TFBSs near enhancer RNA (eRNA) TSSs, enabling a comparison between promoters and enhancers, which were previously suggested to have a unified architecture of initiation regions (Core *et al.* 2014, Andersson *et al.* 2015). The presented tool and resources add complimentary information to existing tools like JASPAR (Castro-Mondragon *et al.* 2022) and PINTS (Yao *et al.* 2022). As combinations of core promoter elements and TFBSs can also be used to enhance gene expression (Juven-Gershon *et al.* 2006), ElemeNT 2023 can be utilized to engineer potent promoters. Therefore, ElemeNT 2023 fills the need for an easy and convenient web-based tool to quickly annotate sequence elements, empowering analysis, quality control and discovery.

## Supplementary Material

btae110_Supplementary_Data
