# Supplementary material for: ElemeNT 2023: an enhanced tool for detection and curation of core promoter elements
Source: Bioinformatics. 2024 Feb 24;40(3):btae110. doi: 10.1093/bioinformatics/btae110 (PMC10950481; doi:10.1093/bioinformatics/btae110)
Supplement: btae110_Supplementary_Data [file btae110_supplementary_data.pdf]

ElemeNT 2023: an enhanced tool for detection and curation of core promoter elements

Orit Adato[1†], Anna Sloutskin[1†], Hodaya Komemi[1], Ian Brabb[2], Sascha Duttke[2], Philipp Bucher[3], Ron Unger[1*] and Tamar Juven-Gershon[1*]

[1]The Mina and Everard Goodman Faculty of Life Sciences, Bar-Ilan University, Ramat Gan, Israel

[2]School of Molecular Biosciences, College of Veterinary Medicine, Washington State University, Pullman, WA, USA

[3]Swiss Institute of Bioinformatics (SIB), Lausanne, Switzerland

*Corresponding authors: The Mina and Everard Goodman Faculty of Life Sciences, Bar-Ilan University, Ramat Gan, Israel. E-mail: ron@biomodel.os.biu.ac.il (R.U.) and tamar.gershon@biu.ac.il (T.J-G.)

[†]These authors contributed equally to this work.

Supplementary material

Contents

ElemeNT 2023: an enhanced tool for detection and curation of core promoter elements 1
- Methods 2
- Supplemental Figures 3
- References 7

Methods

Generation of the CORE file: Promoter sequences (±100bp relative to the transcription start site (TSS)) were downloaded from the Eukaryotic Promoter Database (EPDnew) (Dreos, et al., 2015; Meylan, et al., 2020) at https://epd.expasy.org/epd. ElemeNT 2023 was used to predict the TATA box, dInr, PB and DPE motifs using ElemeNT default parameters settings. The results are depicted in Supplementary Figure 2. Motifs were scanned using a ±10bp window relative to the expected motif location, based on its known distance from the TSS. Result files are available at: https://www.juven-gershonlab.org/resources/core/.

Generation of core promoter element graphs using RAMPAGE analysis: RNA RAMPAGE sequencing data of *Drosophila melanogaster* embryonic development was downloaded from GEO accession number GSE89299. The data included 23 files corresponding to 23 developmental time windows collected from 1 – 23 hours after egg laying. The sequencing data of the first 8 developmental time windows was aligned to *Drosophila melanogaster* genome dm6 using bowtie 2 (Langmead and Salzberg, 2012; Langmead, et al., 2019). TSS peak calling was performed on the output aligned files (bam files) with HOMER v4.11 utility findcsRNATSS.pl (Duttke, et al., 2019). For the identified annotated TSSs, the genomic sequences of the core promoter region (±100 bp around TSS) were downloaded and used as input to ElemeNT to search for the TATA, dInr, PB and DPE motifs. For each of these elements, we used a python script to calculate the fraction (%) of transcripts containing the element identified by ElemeNT at a specific position (out of a total number of the specific element), and their median score in the specific position. The graphs (Supplementary Figure S3) were generated using an R script.

Supplemental Figures

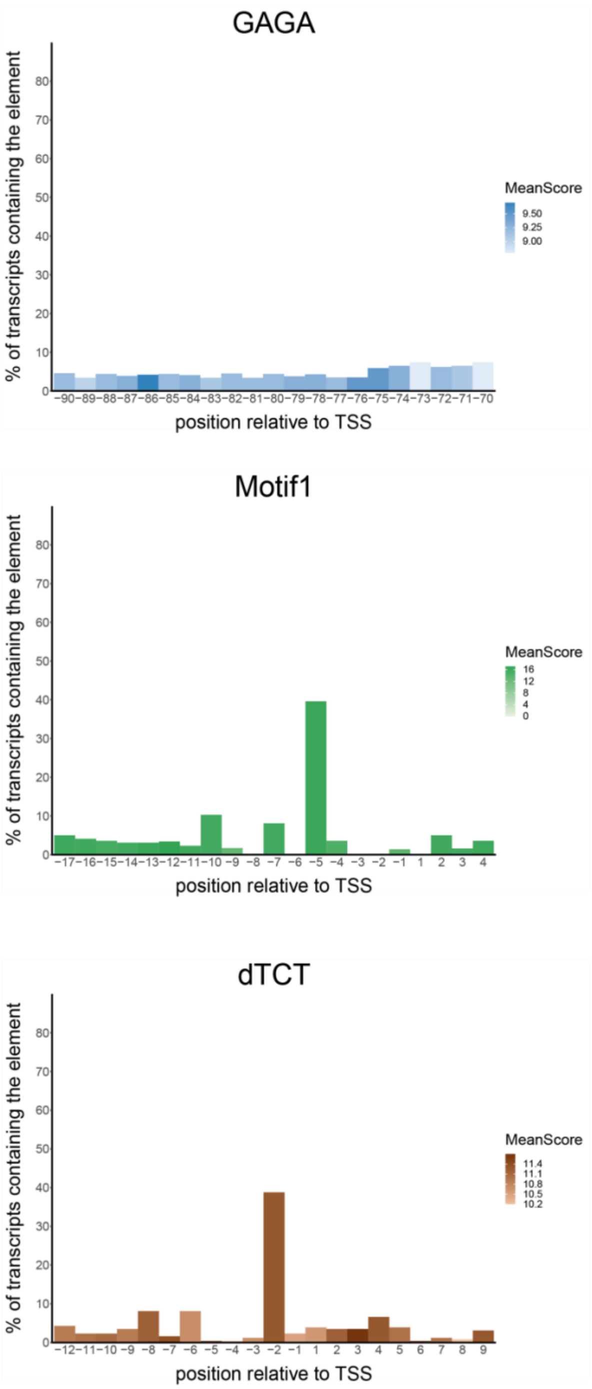

Supplementary Figure S1. Results of ElemeNT run on experimental data of nascent RNA sequencing from the first 8 hours of *Drosophila melanogaster* embryonic development, searching for the GAGA, Motif 1 and dTCT motifs. The X axis represents the predicted element position relative to the TSS, the Y axis represents the percentage of transcripts containing the element at a specific position (out of all transcripts containing the element). The color hue represents the mean score of all predicted elements in the specific position.

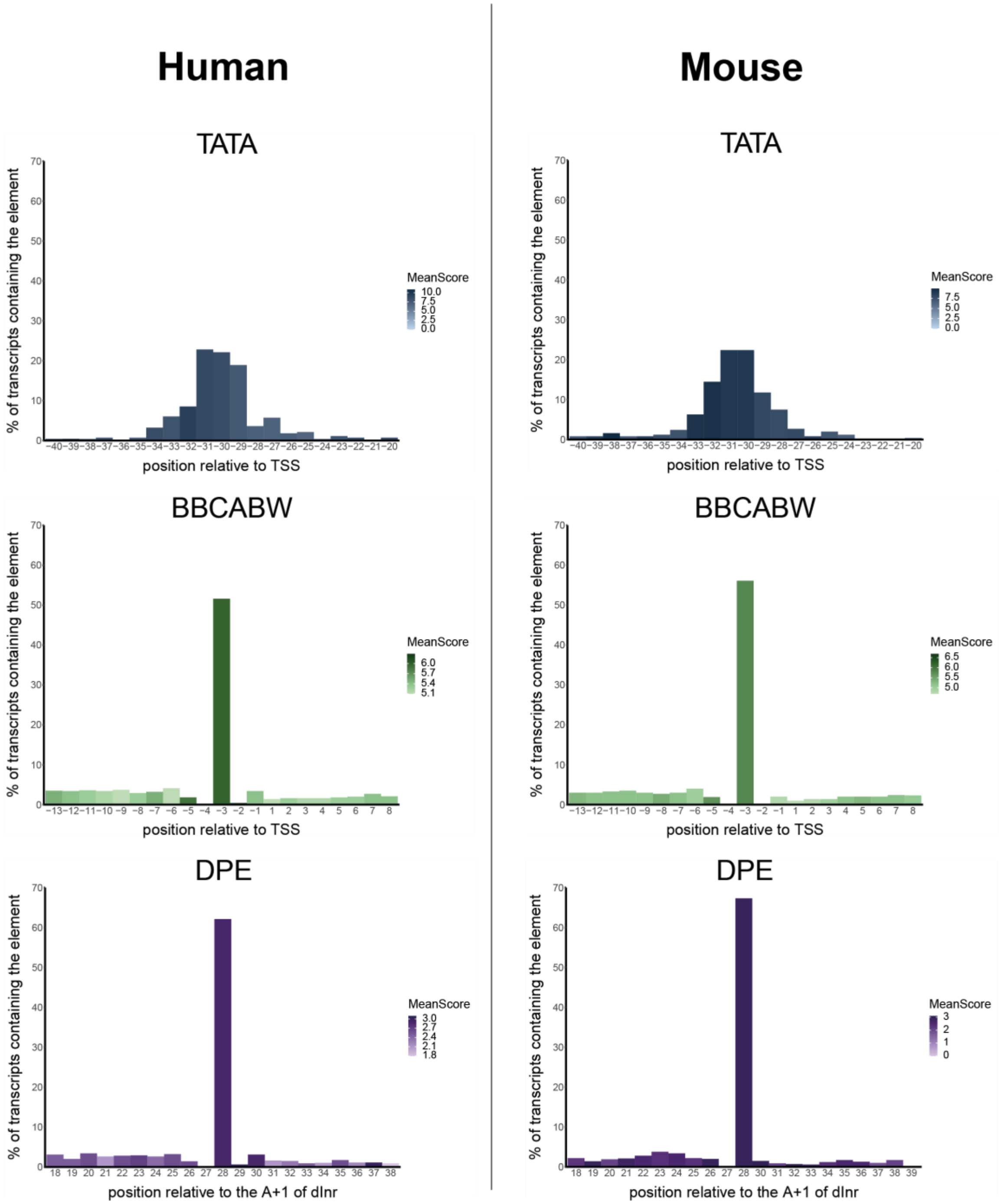

Supplementary Figure S2. Results of ElemeNT 2023 run on TSSs derived from nascent csRNA-seq of human K562 cells and mouse bone marrow-derived macrophages. The X axis represents the predicted element position relative to the TSS for the TATA box, BBCA$_{+1}$BW initiator and the predicted position relative to the A$_{+1}$ of the BBCA$_{+1}$BW initiator for the DPE. The Y axis represents the percentage of transcripts containing the element at the specific position (out of all transcripts containing the element). The color hue represents the mean score of the predicted elements within a specific position, as indicated in the color legend.

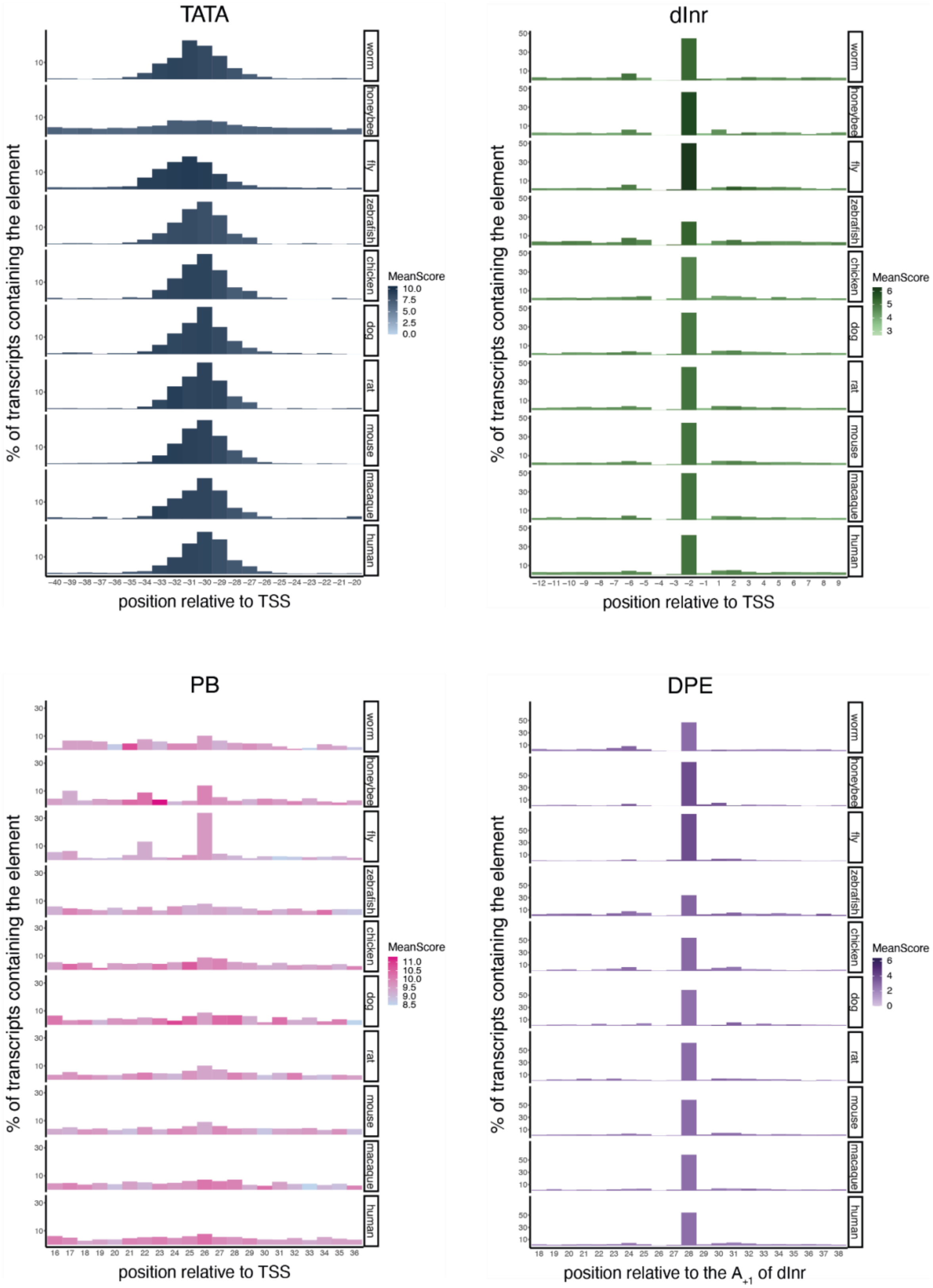

Supplementary Figure S3. Results of ElemeNT run on promoter sequences (±100bp relative to TSS) of 10 different species (*Drosophila melanogaster* (dm6), *Homo sapiens* (hg38),

Macaca mulatta (rheMac8), *Mus musculus* (mm10), *Rattus norvegicus* (rn6), *Gallus gallus* (galGal5), *Canis familiaris* (canFam3), *Apis mellifera* (amel5), *Danio rerio* (danRer7), *Caenorhabditis elegans* (ce6)) downloaded from the Eukaryotic Promoter Database (https://epd.expasy.org/epd). The X axis represents the predicted element position relative to the TSS, the Y axis represents the percentage of transcripts containing the element at the specific position (out of all transcripts of containing the element). The color hue represents the mean score of all predicted elements in the specific position.

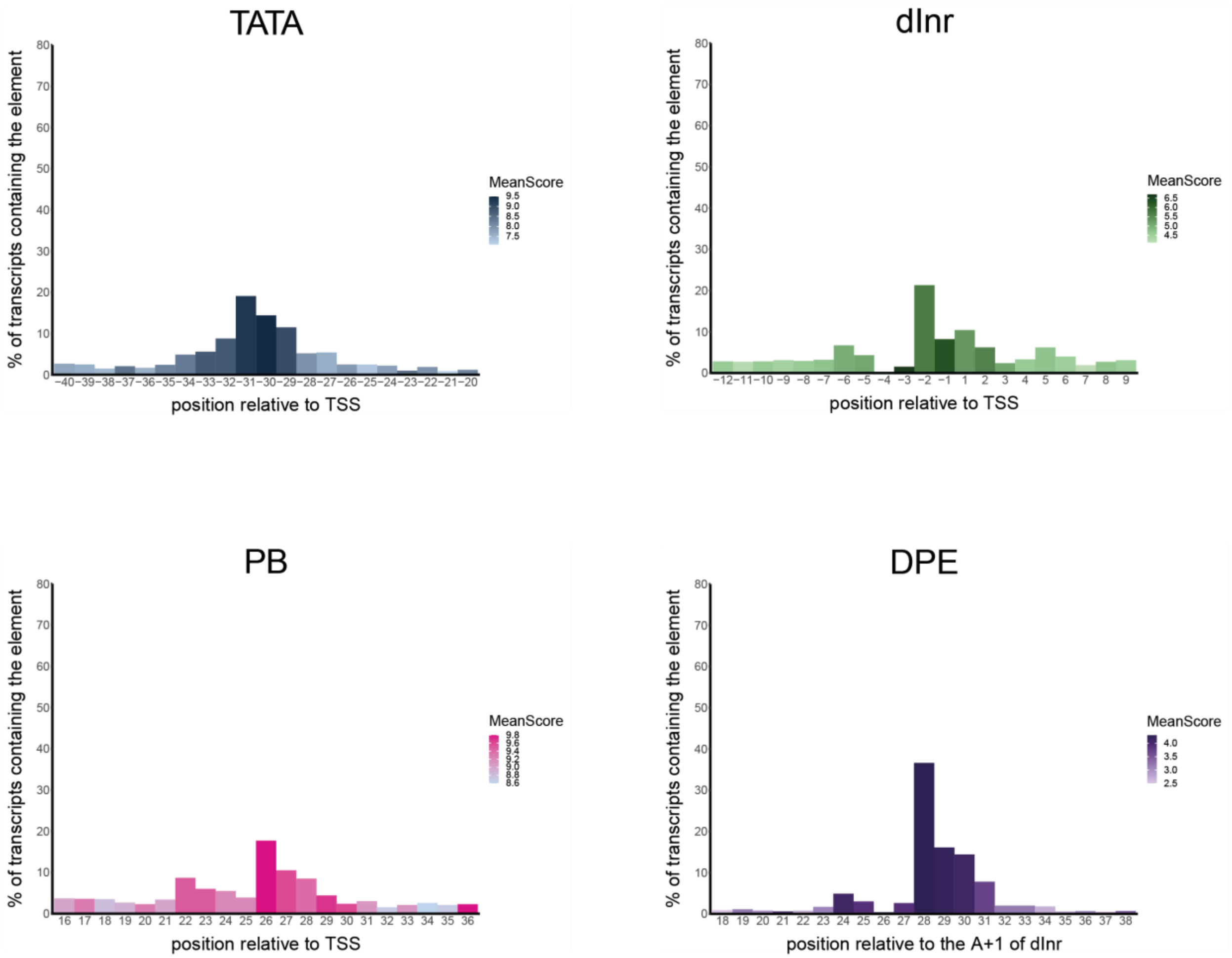

Supplementary Figure S4. Results of ElemeNT run on promoter sequences (±100bp relative to the TSS) on RAMPAGE RNA seq data of 8 developmental windows (corresponding to 8 hours after egg laying). The X axis represents the predicted element position relative to the TSS, the Y axis represents the percentage of transcripts containing the element at the specific position (out of all transcripts of containing the element). The color hue represents the mean score of all predicted elements in the specific position.

References

Dreos, R., *et al.* The Eukaryotic Promoter Database: expansion of EPDnew and new promoter analysis tools. *Nucleic Acids Res* 2015;43(Database issue):D92-96.

Duttke, S.H., *et al.* Identification and dynamic quantification of regulatory elements using total RNA. *Genome Res* 2019;29(11):1836-1846.

Langmead, B. and Salzberg, S.L. Fast gapped-read alignment with Bowtie 2. *Nat Methods* 2012;9(4):357-359.

Langmead, B., *et al.* Scaling read aligners to hundreds of threads on general-purpose processors. *Bioinformatics* 2019;35(3):421-432.

Meylan, P., *et al.* EPD in 2020: enhanced data visualization and extension to ncRNA promoters. *Nucleic Acids Res* 2020;48(D1):D65-D69.
